# Associations of Household Solid Fuel Use With Falls and Fall-Related Injuries in Middle-Aged and Older Population in China: A Cohort Study

**DOI:** 10.3389/ijph.2022.1605425

**Published:** 2023-01-06

**Authors:** Xing Ming, Ruixiao Guo, Mengli Liu, Xiaoman He, Feifei Guo, Shengli Gao

**Affiliations:** ^1^ Pathophysiology Department, School of Basic Medicine, Qingdao University, Qingdao, China; ^2^ Department of Epidemiology and Health Statistics, Public Health College, Qingdao University, Qingdao, China; ^3^ Biomedical Center, Qingdao medical College, Qingdao University, Qingdao, China

**Keywords:** older adults, falls, solid fuels, fall-related injuries, CHARLS

## Abstract

**Objective:** This study evaluated the associations of solid fuels with incidence of falls and fall-related injuries.

**Methods:** Data were taken from wave 1∼4 of the China Health and Retirement Longitudinal Study, including 15,651 participants aged 45 years and older. Modified Poisson regression was used to examine the associations of solid fuels with falls and fall-related injuries.

**Results:** Modified Poisson regression analysis showed that solid fuels users for cooking had an increasing incidence of falls and fall-related injuries, with RR of 1.211 (95% CI: 1.124, 1.305) and 1.248 (95% CI: 1.107, 1.408); for heating had an incidence, with RR of 1.178 (95% CI: 1.062, 1.306) and 1.134 (95% CI: 0.963, 1.335); combined for cooking and heating, with RR of 1.247 (95% CI: 1.105, 1.408) and 1.185 (95% CI: 0.982, 1.431).

**Conclusion:** Our study suggests that solid fuel use is associated with a higher incidence of falls and fall-related injuries among adults aged 45 years and older in China. It is necessary to restrict solid fuel use to reduce household air pollution and make stronger environmental protection policies to improve household environment.

## Introduction

Falls are the second leading cause of accidental injury deaths worldwide; there are approximately 684,000 fatal falls in the world every year, more than 80% of which happen in low- and middle-income countries, usually involving people who are older and more dependent [1]. More than 2.8 million people are treated in emergency departments because of falls every year, and a quarter of falls cause serious injuries, such as fractures or brain injury [2]. Furthermore, older adults who have experienced falls suffer from increased anxiety about falling, physical immobility and loss of independence [3].

Increased rates of falls and fall-related injuries are not the consequence of a single cause but rather the outcome of a range of distinct risk factors, including advancing age, cognitive decline, certain chronic diseases, female gender, medication usage, and environment [4–7]. The underlying biological mechanisms involved in falls and fall-related injuries due to environmental problems remain ill-defined. Studies in animals showed that exposure to biodiesel fuels changed the expression of genes related to antioxidant defense and inflammation [8], which can cause falls [9]. Besides, the study found an increase in glial-fibrillary acidic protein (GFAP) in the corpus callosum of mice exposed to diesel particulate material [10], and long-term exposure to air pollution impaired cognitive function [11–13]. In human studies, it was found that healthy individuals exposed to high levels of air pollutants showed ultrafine particles in the olfactory bulb and α-prominent nuclear proteins in neurons and glial cells, causing a decline in cognitive function [14]. Moreover, it can be seen that air pollution might affect the cognitive function of nervous system, which has close connections with falls [15–18]. Although evidence for a relationship between air pollutants and physical limitations is growing, reliable information on solid fuels and the onset of falls and fall-related injuries is still limited.

A cross-sectional study from China of 12,458 adults reported that solid fuels for cooking may be associated with self-reported and performance-based declines in physical functioning in middle-aged and older populations [19], and a longitudinal study from the Netherlands of 1,763 adults aged 55–85 years reported that performance-based physical functioning decreased with increasing levels of air pollutants [20]. Another nationwide longitudinal study in China, with 10,832 participants (65–105 years of age), concluded that an increase in the annual average concentration of fine particles of 10 μg/m^3^ is accompanied by a 5% reduction in balance ability and that fine particles are also separately associated with various other physical limitations [21].

Based on the China Health and Retirement Longitudinal Study (CHARLS), this study analyzes the impact of household solid fuel use on subsequent falls and fall-related injuries in order to provide a reference for fall prevention and injury reduction among older adults and to improve quality of life in an aging society.

## Methods

### Study Population

The CHARLS is an ongoing nationally representative, longitudinal population-based study of middle-aged and older adults in China. A multistage stratified probability proportional to size sampling design was used to randomly select individuals from 450 villages and resident communities in 150 counties and districts located in 28 provinces.

The baseline survey (wave 1) began in 2011–2012. After baseline, participants were followed up every 2 years for the CHARLS interview. A detailed description of CHARLS can be found in the original article [22]. The biomedical ethics committee of Peking University approved the study, and all study participants signed an informed consent form.

**FIGURE 1:**
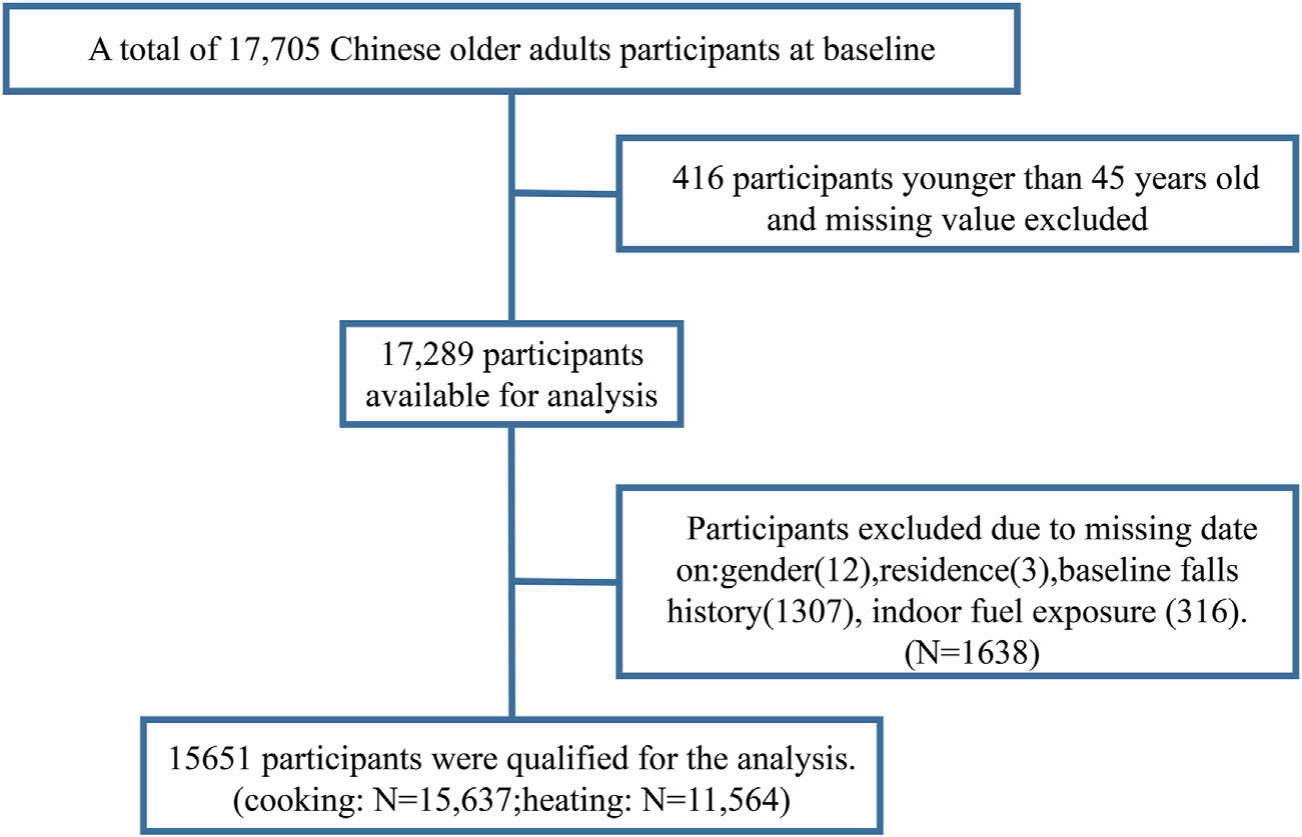
Flow chart of participants (China, 2011).

For the purpose of our analysis, date gathered in 2011/2012 (wave 1) were used as the baseline, with follow-ups in 2013 (wave 2), 2015 (wave 3), and 2018 (wave 4) [22]. We excluded subjects who were younger than 45 years or whose age was missing (*n* = 416) and those with other missing data (e.g., indoor fuel exposure; total *n* = 1,638). A total of 15,651 participants were included in the analysis (Figure 1).

### Household Air Pollution Assessment

According to CHARLS, household energy sources were assessed by a structured questionnaire asking the participants what type of fuel they used. At baseline, participants were classified according to their use of solid or clean fuel based on their responses to the questions “What is the main source of cooking fuel?” and “What is the main heating energy source?” Solid fuel was defined as primary use of coal crop residue, or wood for cooking or heating, and clean fuel use was defined as primary use of solar, natural gas, liquefied petroleum gas or electricity. In order to analyze the impact of solid fuels for cooking heating, or both on falls and fall-related injuries, participants were divided into three categories: 1) those who used clean fuels for both cooking and heating (users of clean fuels), 2) those who used a mixture of solid fuels and clean fuels (e.g., clean fuels to cook but solid fuels to heat) (users of mixed fuels), and 3) those who used solid fuels for both cooking and heating (users of solid fuels) [23].

### Falls and Fall-Related Injuries

A fall is defined as an event which results in a person coming to rest inadvertently on the ground or floor or other lower level [23]. Falls were categorized as either “Yes” or “No,” based on self-reports at baseline and in the follow-up surveys (“Have you fallen down?”). Fall-related injury was identified as injuring seriously enough from falling down to require medical treatment [24]. Determination of fall-related injuries was also categorized based on self-reports at baseline and in the follow-up surveys (“How many times have you fallen down seriously enough to need medical treatment?”) as either “No” (zero fall-related injuries) or “Yes” (one or more fall-related injuries) [25].

### Other Covariates

The covariates in present study were chosen on the basis of previous studies, including gender (female/male), age (continuous), education level (illiterate, primary school and above), marital status (living alone, living with partner), living residence (rural, urban), economic situation (good, fair, poor), smoking status (yes/no), drinking status (yes/no), body mass index (BMI) (continuous), physical activity (vigorous, moderate, light, or insufficient), self-reported previous diseases including hypertension (yes/no), diabetes (yes/no), stroke (yes/no), psychiatric problems (yes/no) and memory-related diseases (yes/no) obtained by uniformly trained investigators through a standard questionnaire.

The education level of the participants was obtained by asking the question “What is the highest level of education completed?”. Educational level was categorized as “illiterate” (no formal education illiterate) and “primary school and above” (did not finish primary school but capable of reading or writing, home school, elementary school, middle school, high school, vocational school, Two/Three Year College/Associate degree, Four Year College/Bachelor’s degree, Master’s degree, Doctoral degree/Ph.D.) [23, 26]. Participants who were separated, divorced, widowed or never married were coded as “living alone” group, while those who were married or partnered were coded as “living with partner” group [27]. Self-reported family economic status “very high” and “relatively high” were combined as “good”, “average” still is “fair”, whereas “relatively poor” and “poor” were combined as “poor” [28]. Smoking was defined as a person having smoked more than the equivalent of 100 cigarettes during their life, including cigarettes, pipe tobacco, and chewing tobacco. Non-drinkers were those who had drunk no alcoholic beverages in the previous year [23]. BMI was calculated as weight in kilograms divided by height in meters squared.

### Statistical Analysis

First, the participants were grouped according to whether they had experienced a fall and whether they had experienced a fall-related injury. Continuous variables were described as mean ± standard deviation, and dichotomous variables were described as frequency (percentages). Univariate analysis were used to estimate risk ratio and the associated 95% confidence intervals (CI).

Second, modified Poisson regression analysis was used to evaluate solid fuels as a risk factor for falls and fall-related injuries. To test the stability of the modified Poisson regression model, a set of models were used in the current study: The cooking-related, heating-related, and mixed-fuels analyses were performed with no adjustment (model 1), then with adjustments for age and sex at baseline (model 2), and finally with adjustments for age, sex, economic situation, education, marital status, residence, smoking, drinking, BMI, physical activity, hypertension, diabetes, stroke, psychiatric problems, memory-related diseases and baseline falls history (model 4).

Third, subgroup analyses were conducted, stratified by sex and area of residence. Corresponding variable was excluded in the subgroup analyses. The results are presented as risk ratios (RR) with 95% confidence intervals (CI). Falls and fall-related injuries were compared between clean fuels, mixed fuels, and solid fuels, with clean fuels used as the reference category. SPSS version 25.0 was used for all analyses.

## Results

### Participant Characteristics

Table 1 shows the characteristics of the study participants. CHARLS had a high response rate of 80.5%, which further enhances the reliability and representativeness of the study (The overall response rate were 80.51% in 2011, 82.63% in 2013, 82.13% in 2015, 83.84% in 2018.). A total of 15,651 participants aged 45 or over were included in this study at baseline, of whom 36.0% had experienced falls and 17.7% had suffered fall-related injuries, which is similar to the proportion of the elderly who sought medical treatment after falling in previous studies [29]. The elderly female is slightly higher than the elderly male and the education level is generally low. The distribution of these basic characteristics is basically consistent with the survey data of the National Bureau of Statistics of China. These indicate that the respondents selected in this paper are representative. Compared with participants who without falls and fall-related injuries, participants who had falls and fall-related injuries were older, more likely to be single, lower education levels, be smokers, live rurally, and use solid fuels. In the univariate analysis, sex, age, education level, marital status, residence, economic situation, smoking, drinking, BMI, physical activity, hypertension, diabetes, stroke, psychiatric problems, memory-related diseases and baseline falls history. Hypertension had statistical significance among the elderly.

**TABLE 1:** The characteristics of the study participants according to household fuels use type in baseline (China, 2011).

Characteristics	Total	Falls			Fall, related injuries		
		Yes	No	Unadjusted relative risk (95% CI)	Yes	No	Unadjusted relative risk (95% CI)
		N = 5,273 (36.0%)	N = 9,372 (64.0%)		N = 2,592 (17.7%)	N = 12,051 (82.3%)	
Sex							
Female	8,189 (52.3%)	3,117 (59.1%)	4,585 (48.9%)	1.0	1,636 (63.1%)	6,064 (50.3%)	1.0
Male	7,462 (47.7%)	2,156 (40.9%)	4,787 (51.1%)	0.767 (0.734, 0.802)	956 (36.9%)	5,987 (49.7%)	0.648 (0.603, 0.697)
Age, years	59.45 ± 9.754	58.41 ± 9.312	60.54 ± 9.7820	1.015 (1.012, 1.017)	50.94 ± 9.809	58.80 ± 9.436	1.010 (1.015, 1.022)
Education							
Illiterate	4,297 (27.5%)	1722 (32.7%)	2,297 (24.5%)	1.0	947 (36.5%)	3,070 (25.5%)	1.0
Primary school and above	11,354 (72.5%)	3,551 (67.3%)	7,075 (75.5%)	0.780 (0.746, 0.816)	1,645 (63.5%)	8,981 (74.5%)	0.657 (0.612, 0.705)
Marital status							
Living with partner	13,633 (87.1%)	4,454 (84.5%)	8,391 (89.5%)	1.0	2,155 (83.1%)	10,688 (88.7%)	1.0
Living alone	2018 (12.9%)	819 (15.5%)	981 (10.5%)	1.312 (1.241, 1.388)	437 (16.9%)	1,363 (11.3%)	1.447 (1.322, 1.583)
Residence							
Rural	11,981 (76.6%)	4,317 (81.9%)	7,143 (76.2%)	1.0	2,187 (84.4%)	9,271 (76.9%)	1.0
Urban	3,670 (23.4%)	956 (18.1%)	2,229 (23.8%)	0.797 (0.752, 0.844)	405 (15.6%)	2,780 (23.1%)	0.666 (0.604, 0.735)
Economic situation							
Good	464 (3.0%)	133 (2.5%)	289 (3.1%)	1.0	70 (2.7%)	352 (3.0%)	1.0
Fair	8,191 (52.9%)	2,512 (48.13%)	5,164 (55.7%)	1.038 (0.899, 1.199)	1,235 (48.1%)	6,441 (54.0%)	0.970 (0.778, 1.209)
Poor	6,817 (44.1%)	2,581 (49.4%)	3,810 (41.1%)	1.281 (1.110, 1.479)	1,264 (49.2%)	5,125 (43.0%)	1.193 (0.958, 1.486)
Smoking							
No	9,500 (60.7%)	3,419 (64.8%)	5,509 (58.8%)	1.0	1759 (67.9%)	7,167 (59.5%)	1.0
Yes	6,147 (39.3%)	1854 (35.2%)	3,860 (41.2%)	0.847 (0.809, 0.887)	833 (32.1%)	4,881 (40.5%)	0.740 (0.686, 0.798)
Drinking							
No	10,557 (67.5%)	3,653 (69.3%)	6,186 (66.0%)	1.0	1825 (70.4%)	8,012 (66.5%)	1.0
Yes	5,090 (32.5%)	1,620 (30.7%)	3,183 (34.0%)	0.908 (0.867, 0.952)	767 (29.6%)	4,036 (33.5%)	0.861 (0.797, 0.930)
Physical activity							
Insufficient	757 (11.3%)	258 (11.2%)	410 (10.3%)	1.0	121 (10.8%)	547 (10.6%)	1.0
Light	1,649 (24.7%)	524 (22.8%)	979 (24.6%)	0.970 (0.895, 1.051)	261 (23.2%)	1,242 (24.1%)	0.939 (0.824, 1.070)
Moderate	1988 (12.7%)	685 (29.8%)	1,199 (30.2%)	0.930 (0.852, 1.015)	330 (29.3%)	1,554 (30.2%)	0.931 (0.809, 1.071)
Vigorous	2,284 (14.6%)	830 (36.1%)	1,384 (34.8%)	1.030 (0.923, 1.015)	413 (36.7%)	1801 (35.0%)	0.971 (0.808, 1.166)
BMI	23.488 ± 3.924	23.415 ± 3.948	23.573 ± 3.884	0.993 (0.987, 0.999)	23.262 ± 3.742	23.572 ± 3.942	0.983 (0.973, 0.993)
Hypertension							
No	11,707 (75.2%)	3,875 (74.0%)	7,155 (76.7%)	1.0	1901 (73.7%)	9,128 (76.1%)	1.0
Yes	3,862 (24.8%)	1,365 (26.0%)	2,174 (23.3%)	1.098 (1.046, 1.153)	677 (26.3%)	2,861 (23.9%)	1.110 (1.026, 1.202)
Diabetes							
No	14,591 (94.1%)	4,862 (93.1%)	8,812 (94.9%)	1.0	2,422 (94.2%)	11,250 (94.3%)	1.0
Yes	913 (5.9%)	359 (6.9%)	9,285 (5.1%)	1.214 (1.119, 1.316)	150 (5.8%)	682 (5.7%)	1.018 (0.877, 1.182)
Stroke							
No	15,256 (97.7%)	5,104 (97.1%)	9,186 (98.2%)	1.0	2,526 (97.7%)	11,763 (97.9%)	1.0
Yes	356 (2.3%)	152 (2.9%)	166 (52.2%)	1.338 (1.191, 1.504)	60 (2.3%)	257 (2.1%)	1.071 (0.850, 1.348)
Psychiatric problems							
No	15,400 (98.9%)	5,175 (98.6%)	9,238 (99.0%)	1.0	2,542 (98.6%)	11,869 (98.9%)	1.0
Yes	178 (1.1%)	74 (1.4%)	89 (1.0%)	1.264 (1.067, 1.498)	37 (1.4%)	126 (1.1%)	1.287 (0.967, 1.712)
Memory-related diseases							
No	15,393 (98.7%)	5,167 (98.4%)	9,252 (99.1%)	1.0	2,543 (98.5%)	11,874 (98.9%)	1.0
Yes	203 (1.3%)	86 (1.6%)	87 (0.9%)	1.387 (1.192, 1.614)	38 (1.5%)	135 (1.1%)	1.245 (00.938, 1.653)
Baseline falls history							
No	13,158 (84.1%)	3,908 (74.1%)	8,402 (89.7%)	1.0	1888 (72.8%)	10,420 (86.5%)	1.0
Yes	2,493 (15.9%)	1,365 (25.9%)	970 (10.3%)	1.841 (1.764, 1.922)	704 (27.2%)	1,631 (13.5%)	1.965 (1.825, 2.117)
Baseline fall-related injuries							
No	14,239 (91.1%)	4,510 (85.7%)	8,820 (94.2%)	1.0	2,133 (82.6%)	11,195 (93.0%)	1.0
Yes	1,392 (8.9%)	750 (14.3%)	545 (5.8%)	1.712 (1.625, 1.803)	450 (17.4%)	845 (7.0%)	2.171 (1.996, 2.362)
Cooking							
Clean fuel	7,157 (45.8%)	2072 (39.3%)	4,489 (47.9%)	1.0	993 (38.4%)	5,568 (46.2%)	1.0
Solid fuel	8,480 (54.2%)	3,195 (60.7%)	4,876 (52.1%)	1.253 (1.199, 1.311)	1,595 (61.6%)	6,474 (53.8%)	1.306 (1.215, 1.404)
Heating							
Clean fuel	3,009 (26.0%)	857 (21.7%)	1895 (27.3%)	1.0	415 (21.2%)	2,337 (26.2%)	1.0
Solid fuel	8,555 (74.0%)	3,100 (78.3%)	5,042 (72.7%)	1.223 (1.149, 1.301)	1,541 (78.8%)	6,599 (73.8%)	1.255 (1.137, 1.387)
Combined cooking and heating							
Clean fuel	2,438 (21.1%)	1,556 (22.5%)	660 (16.7%)	1.0	323 (16.5%)	1893 (21.2%)	1.0
Mixed fuel	2,703 (23.4%)	882 (22.3%)	1,680 (24.2%)	1.156 (1.063, 1.256)	432 (22.1%)	2,130 (23.9%)	1.157 (1.013, 1.321)
Solid fuel	6,409 (55.5%)	2,409 (61.0%)	3,694 (53.3%)	1.325 (1.234, 1.423)	1,197 (61.3%)	4,904 (54.9%)	1.346 (1.202, 1.507)

### Relationships Between Solid Fuels, Falls, and Fall-Related Injuries

Table 2 indicates that in the unadjusted model (model 1), the age- and sex-adjusted (model 2), and the model 3, the use of solid fuels was significantly associated with the incidence of falls and fall-related injuries. After smoking, dringing, BMI, physical activity, hypertension, diabetes, stroke, psychiatric problem, memory-related diseases and baseline falls history were added into model 4, the results show that who used solid fuels for cooking had an increased incidence of falls (RR = 1.211; 95% CI: 1.124–1.305) and fall-related injuries (RR = 1.248; 95% CI: 1.107–1.408); those who used solid fuels for heating also had an incidence of falls (RR = 1.178; 95% CI: 1.062–1.306) and fall-related injuries (RR = 1.134; 95% CI: 0.963–1.335). Those who combined used solid fuels for heating and cooking had an incidence of falls (RR = 1.247; 95% CI: 1.105–1.408) and fall-related injuries (RR = 1.185; 95% CI: 0.982–1.431).

**TABLE 2:** Relative risk for incidence of falls events and fall-related injuries by primary fuel exposure (China, 2011–2018).

	Valid n	Model 1	Model 2	Model 3	Model 4
		RR (95% CI)	RR (95% CI)	RR (95% CI)	RR (95% CI)
Falls					
Cooking	5,267				
Clean fuel	2072	(reference)	(reference)	(reference)	(reference)
Solid fuel	3,195	**1.253 (1.199,1.311)**	**1.253 (1.199,1.311)**	**1.252 (1.197,1.309)**	**1.211 (1.124,1.305)**
Heating	3,957				
Clean fuel	857	(reference)	(reference)	(reference)	(reference)
Solid fuel	3,100	**1.223 (1.149,1.301)**	**1.223 (1.149,1.301)**	**1.218 (1.145,1.297)**	**1.178 (1.062,1.306)**
Combined cooking and heating	3,951				
Clean fuel	660	(reference)	(reference)	(reference)	(reference)
Mixed use clean fuel and solid fuel	882	**1.156 (1.063, 1.256)**	**1.156 (1.063, 1.256)**	**1.153 (1.060, 1.254)**	1.112 (0.968, 1.227)
Solid fuel	2,409	**1.325 (1.234, 1.423)**	**1.325 (1.234, 1.423)**	**1.321 (1.230, 1.419)**	**1.247 (1.105, 1.408)**
Fall-related injuries					
Cooking	2,588				
Clean fuel	993	(reference)	(reference)	(reference)	(reference)
Solid fuel	1,595	**1.306 (1.215, 1.404)**	**1.306 (1.215, 1.404)**	**1.303 (1.212, 1.401)**	**1.248 (1.107, 1.408)**
Heating	1956				
Clean fuel	415	(reference)	(reference)	(reference)	(reference)
Solid fuel	1,541	**1.255 (1.137, 1.387)**	**1.255 (1.137, 1.387)**	**1.255 (1.135, 1.387)**	1.134 (0.963, 1.335)
Combined cooking and heating	1952				
Clean fuel	323	(reference)	(reference)	(reference)	(reference)
Mixed use clean fuel and solid fuel	432	**1.157 (1.013, 1.321)**	**1.157 (1.013, 1.321)**	**1.155 (1.011, 1.320)**	1.008 (0.982, 1.431)
Solid fuel	1,197	**1.346 (1.202, 1.507)**	**1.346 (1.202, 1.507)**	**1.345 (1.201, 1.507)**	1.185 (0.982, 1.431)

Boldface indicates significance.

Model 1 was unadjusted.

Model 2 was adjusted for age, sex.

Model 3 was adjusted for Model 2 + education, marital status, economic situation, residence.

Model 4 was adjusted for Model 3 + smoking drinking, BMI, physical activity, hypertension, diabetes, stroke, psychiatric problems, memory-related diseases and baseline falls history.

### Association of Solid Fuels With Falls and Fall-Related Injuries Stratified by Sex and Living Residence

Table 3 displays the results from modified Poisson regression stratified by sex and area of residence. When Combined cooking and heating, solid fuels were significantly associated with falls among female [relative ratio (RR) 1.242, 95% CI: 1.070–1.441], male [(RR) 1.262, 95% CI: 1.029–1.548] and rural [(RR) 1.184, 95% CI: 1.027–1.365). No significant effect modification by sex (P - interaction = .558) and area of residence (P - interaction = 0.983) were observed.

**TABLE 3:** Subgroup analyses on the associations of household solid fuels for cooking, heating and combined cooking and heating with incidence of falls events and fall-related injuries (China, 2011–2018).

	Falls	*P* for interaction	Fall-related injuries	*P* for interaction
	RR (95%CI)		RR (95%CI)	
Cooking				
Gender^a^		0.604		0.276
Male	**1.244 (1.098, 1.408)**		**1.146 (0.938, 1.400)**	
Female	**1.194 (1.089, 1.308)**		**1.317 (1.134, 1.529)**	
Area of residence^b^		0.870		0.328
Urban	1.133 (0.893, 1.438)		0.899 (0.565, 1.429)	
Rural	**1.158 (1.064, 1.259)**		**1.144 (1.001, 1.307)**	
Heating				
Gender^a^		0.872		0.893
Male	1.168 (0.984, 1.386)		1.121 (0.853, 1.473)	
Female	**1.189 (1.045, 1.352)**		1.147 (0.936, 1.406)	
Area of residence^b^		0.877		0.825
Urban	1.153 (0.895, 1.484)		1.1.7 (0.721, 1.699)	
Rural	**1.128 (1.004, 1.267)**		1.050 (0.876, 1.258)	
Combined cooking and heating				
Gender^a^		0.558		0.254
Male				
Mixed use clean fuels and solid fuels	1.200 (0.955, 1.508)		1.122 (0.787, 1.600)	
Solid fuel	**1.262 (1.029,1.548)**		1.108 (0.807, 1.521)	
Female				
Mixed use clean fuels and solid fuels	1.062 (0.893, 1.263)		0.944 (0.714, 1.247)	
Solid fuel	**1.242 (1.070,1.441)**		1.236 (0.979, 1.561)	
Area of residence^b^				
Urban		0.983		0.875
Mixed use clean fuels and solid fuels	1.062 (0.797, 1.415)		0.863 (0.525, 1.414)	
Solid fuel	1.194 (0.862, 1.655)		1.094 (0.631, 1.895)	
Rural				
Mixed use clean fuel and solid fuel	1.087 (0.925, 1.277)		0.980 (0.763, 1.257)	
Solid fuel	**1.184 (1.027,1.365)**		1.082 (0.871, 1.343)	

Boldface indicates significance.

^a^ Model was adjusted for age, education, marital status, economic situation, residence, smoking drinking, BMI, physical, activity, hypertension, diabetes, stroke, psychiatric problems, memory-related diseases and baseline falls history.

^b^ Model was adjusted for age, sex, education, marital status, economic situation, smoking drinking, BMI, physical, activity, hypertension, diabetes,stroke, psychiatric problems, memory-related diseases and baseline falls history.

## Discussion

Falls and fall-related injuries are notable problems in the healthcare industry, especially for patients and older people. Risk factors for falls include muscle weakness, agitation, confusion, postural hypotension, and sedative medication [30]. Recent studies have shown that environmental pollutants such as PM2.5 are also risk factors for falls and fall-related injuries [31]. Short-term and long-term exposure to environmental pollutants may be associated with a series of adverse health outcomes of physical function, such as slower gait, balance disorders, and tremors [14, 21]. Moreover, some studies have shown the relationships between the indoor use of solid fuels and respiratory diseases, cardiovascular diseases, and cognitive dysfunction in different regions [32–34]. Although outdoor air pollution has been linked to falls, but limited studies have investigated the relationship between household air pollution of solid fuels and falls and fall-related injuries in adults. Therefore, we analyzed data from the China Health and Retirement Survey (CHARLS), a national survey of about 17,000 residents aged over 45 to explore the associations between solid fuels with falls and fall-related injuries.

In this large, national, prospective cohort study in China, we found that when the covariates were adjusted—gender, age education level, marital status, living residence, economic situation, smoking status, drinking status, body mass index, physical activity, self-reported previous diseases including hypertension, diabetes, stroke, psychiatric problems and memory-related diseases and baseline falls history—the use of solid fuels for cooking and heating at baseline increased the risks of falls and fall-related injuries.

Our findings showed that solid fuels used for cooking were significantly correlated with falls and fall-related injuries among adults over 45 years old, which possibly suggested that solid fuels might be a risk factor for falls and fall-related injuries. These are consistent with the results of several previous studies on the decline of physical functions of participants exposed to outdoor air pollution. A study from Netherlands suggests that exposure to air pollution may adversely affect physical performance of older adults [20]. Similarly, another prospective cohort study with 6,157 participants (≥65 years of age) from Chicago, Illinois, USA indicated that long-term exposure to nitrogen oxides (NOx) may be associated with a faster aging-related decline in physical functioning [35].

In addition, the proportion of solid fuels used for heating is over 70% in Table 1, which is consistent with the increase in the stocking of domestic fuel for winter heating in northern China during the same period [36]. However, our results suggested that in the full model, solid fuels used for heating was significantly correlated with falls rather than fall-related injuries among adults over 45 years old. Such results may be caused by a large number of exposure misclassification caused by factors such as ventilation, emissions from neighbor stove and habits [37]. Additionally, due to concerns about carbon monoxide poisoning, people often pay more attentions to ventilation when heating than when cooking, potentially reducing the levels of pollutants produced by heating fuel use [4].To analyze whether a higher proportion of use of solid fuels induces more falls and fall-related injuries and to study the combined effect of solid fuels used for cooking and heating on falls and fall-related injuries, we combined the data for fuels used for cooking with those used for heating and divided the participants into three categories: the users of clean fuels, the users of mixed fuels, and the users of solid fuels. The results suggested that indoor users of combined cooking and heating solid fuels almost had more falls rather than fall-related injuries. Dual exposure to indoor air pollution from cooking and heating may result in overlapping effects and constitute a persistent danger to human health [23]. These results might provide new knowledge about the impact of solid fuel use on humans that includes depression, sleep disturbance, arthritis, COPD, and so on [5, 23, 38, 39].

Subgroup analysis suggested that the associations between solid fuels with falls and fall-related injuries were significant in almost all different subgroups categorized by sex and residence. This finding confirmed our main results. Compared with these previous studies, this study not only had much larger sample size over a wider age range (≥45 years old at baseline) and a longer follow-up time, but also paid attention to the type of solid fuel use on falls and injurious falls. The underlying biological mechanisms involved in the increased likelihood of falls and fall-related injuries due to household solid fuel use remain ill defined. Numerous risk factors for falls such as psychomotor retardation (including, cognitive problems, dementia, and so on) and gait alteration are common in older people those who were exposure in air pollution [40]. Individuals who were suffered in air pollution also tend to exhibit cognitive deficits [11] that affect attention, executive functions, and processing speed [15, 41], which can all increase the risk of falls and injurious falls. Prior studies supported that people with diabetes were at a significantly higher risk of falls and injurious falls than people without diabetes across [42]. Huiyu Wang et al. found that each 10 μg/m3 increase in annual averaged concentrations of PM2.5 was associated with a 5% risk of reduced balance ability [21]. In addition, some experiments have demonstrated the adverse effects of solid fuels or indoor air pollution on physical health. A cross-sectional study showed that household use of solid fuels is also a risk factor for the development of cognitive impairment [4]. Also, exposure to indoor PM2.5 can increase IL-7 levels in C57 mice [43], and elevated IL-7 levels increased the likelihood of single and recurrent falls [44]. In general, the adverse effect of solid fuels on health might aggravate the diseases that induce falls and fall-related injuries.

Our study has several strengths. First, the main strength of the present study is that it is a national population-based sample, providing rich data to support the reliability of our conclusions. Second, our study simultaneously investigated the relationship between solid fuels and falls and fall-related injuries, and prospectively assessed the correlation. Finally, the national representation of CHARLS is widely recognized, so our results can be used not only as evidence for fall prevention among older people in China, but also as a reference for future research in other countries, especially in developing countries.

However, there are also some limitations in this study. First, the current study adopted modified Poisson regression rather than survival analysis because falls are investigated every 2 years, and we cannot obtain accurate exposure time from the questionnaire. Second, despite controlling for many potential covariates, residual confounding may have influenced our observed relationships. Third, information collected from participants was based on self-report, so this study cannot rule out the possibility of recall bias. Previous research consistently showed that older adults tended to under-report falls because they did not recognize the severity of a fall or did not remember a fall with less severe consequences [45]. Therefore, there might be recall bias. Fourth, CHARLS has amounts of missing or incomplete data. Incomplete datasets may lead to potential biases. Finally, due to the true quantitative value of pollutant concentration is not involved in this questionnaire, we can’t directly explore the relationship between the real value caused by household air pollution with falls and fall-related injuries in middle-aged and older people. Although the type of fuels has adopted to replace it, which greatly weakens the causality of this study. It is expected that subsequent studies will be conducted on pollutant types or specific concentrations. However, we hope this study may generate people’s interest in gaining a better understanding of the relationships between solid fuels and falls or fall-related injuries.

### Conclusion

In conclusion, we identified that household solid fuel use is closely associated with falls and fall-related injuries. Strategies that educate individuals on the efforts to reduce household solid fuel use should be established, which is significant to effectively prevent falls and fall-related injuries.
